# Translation, Cultural, and Clinical Validation of the Lithuanian Version of the Spiritual Needs Questionnaire among Hospitalized Cancer Patients

**DOI:** 10.3390/medicina55110738

**Published:** 2019-11-14

**Authors:** Olga Riklikienė, Lina Spirgienė, Snieguolė Kaselienė, Žydrūnė Luneckaitė, Jūratė Tomkevičiūtė, Arndt Büssing

**Affiliations:** 1 Faculty of Nursing, Medical Academy, Lithuanian University of Health Sciences, A. Mickevičiaus str. 9, LT44307 Kaunas, Lithuania; lina.spirgiene@lsmuni.lt; 2 Faculty of Public Health, Medical Academy, Lithuanian University of Health Sciences, A. Mickevičiaus str. 9, LT44307 Kaunas, Lithuania; snieguole.kaseliene@lsmuni.lt (S.K.); zydrune.luneckaite@lsmuni.lt (Ž.L.); 3 Faculty of Medicine, Medical Academy, Lithuanian University of Health Sciences, A. Mickevičiaus str. 9, LT44307 Kaunas, Lithuania; jurate.tomkeviciute@lsmuni.lt; 4 Professorship Quality of Life, Spirituality and Coping, Faculty of Health, Witten/Herdecke University, Gerhard Kienle Weg 4, 58313 Herdecke, Germany; a.buessing@iunctus.de

**Keywords:** cancer patients, Lithuania, psychometrics, spiritual needs, translation

## Abstract

*Background and Objectives*: The aim was to translate and validate the spiritual needs questionnaire for its use in the Lithuanian context. *Materials and Methods*: A descriptive, cross-sectional survey design was applied. Structural individual interview method (face-to-face) was employed to collect data on spiritual needs of cancer patients. Responses were obtained from 247 patients hospitalized in nursing and supportive treatment units at public hospitals. Data were analyzed using the Statistical Package for Social Sciences (IBM SPSS Statistics) version 22.0. To assess the psychometric properties of the scale, Cronbach’s alpha, split half test, average inter-item, and item-total correlations were calculated for internal consistency. Exploratory factor analysis was used to confirm the construct validity of the translated version of instrument. *Results*: Lithuanian version of The Spiritual Needs Questionnaire (27 items) had a good internal consistency (Cronbach’s alpha = 0.94). The existential and connectedness with family needs factor had the lowest Cronbach’s alpha (0.71) in relation to other factors: Religious needs (0.93), giving/generativity and forgiveness needs (0.88), and inner peace needs (0.74). Split-half test showed strong relationship between the both halves of the test. The item difficulty (1.47 (mean value)/3) was 0.49; while all values were in acceptable range from 0.20 to 0.80. Item-total correlations were inspected for the items in each of the four SpNQ-27 factors. *Conclusions*: The Lithuanian version of Spiritual needs questionnaire demonstrated adequate psychometric properties of the instrument. This instrument, as a screening tool and conversational model, is recommended for clinicians in health care practice to identify patients with spiritual needs.

## 1. Introduction

Spirituality in healthcare is gaining increasing attention as the care paradigm has moved (at least in theory) from biomedical towards a holistic approach, which includes all dimensions and needs of patients and their family. Serious illness creates vulnerable conditions of insecurity, fears, and worries about the course of disease and life concerns that raises fundamental psychosocial, existential, and spiritual issues for patients. Physicians, together with other professionals of healthcare, of course, have the duty to care for a person’s health and restoration of symptoms on the one hand. However, on the other hand—as part of a comprehensive healthcare concept—they should also consider addressing patients’ unmet spiritual concerns through providing spiritual care on the basis of legislation, ethical codes, and on research evidence [1]. Despite this understanding, in clinical practice, clinicians caring for cancer patients are firstly focused on medical diagnosis and clinical nursing problems, by prescribing treatment and medications—and often fail to address patients’ spiritual needs and/or identify signs of spiritual distress [2].

Even if medical staff identify patients’ spiritual concerns as an equal, specialized domain of care [3], one may ask how much spiritual care can be provided in a busy clinical setting without enough staff, missing spiritual care standard, and lacking appropriate preparation to assess patients’ spirituality. Untrained in spiritual assessment and counselling, clinicians are not able to design and implement a plan spiritual care, and thus they put off this task to clergy staff. Although this may be appropriate for religious patients, in today’s secularized societies, a majority of patients reject institutionalized religiosity with its rituals and practices, claiming themselves either as persons with a more universal spirituality or as religious persons/non-believers. Moreover, according to Sulmasy [4] those who consider themselves spiritual but not religious will also have genuine spiritual needs that are as significant as those of religious patients. However, spiritual needs of non-religious/non-spiritual persons (R−S−) are quantitatively and qualitatively different from that of religious or spiritual persons [5,6]. In relation to healthcare service, there is evidence that advanced cancer patients who report insufficiently supported individual religious and/or spiritual needs have increased costs of care [7], because those lacking adequate spiritual care are treated in a more expensive intensive care unit, while those with spiritual care support are more often treated in hospice or long-term nursing care facilities. Additionally, the patient’s (as well as the physician’s) religion affiliation is associated with his/her end-of-life care decisions about withdrawing/withholding of life sustaining treatment in intensive care unit [8]. This underlines that ignoring a person’s spiritual concerns and needs and other humanistic aspects of care not only disrespects individual patients, but also discredits the organization of care—and requires changes in the healthcare professional curricula [3,9,10].

For conceptual reasons, in healthcare research, one should differentiate the terms ‘spiritual’ and ‘religious’, implying that a person may see him/herself as spiritual but not religious (R−S+), as religious but not spiritual (R+S−), as both religious and spiritual (R+S+), or as neither religious nor spiritual (R−S−) [11,12]. Both the interpretation of personal spiritual concerns and the extent of one’s spiritual needs might be different. Spiritual needs are a multidimensional phenomenon, which is dependent on religious and cultural background. For patients and caregivers, it is sometimes difficult to define what spiritual needs and what spiritual care does mean for them. Spiritual needs of cancer patients may have a wide spectrum of queries starting with religious rituals and talks with faith community members, followed by the relationship with family members and friends; gratitude, hope, and forgiveness; loving others; particular connection with nature in a religious or nonreligious way; or deep self-assessment and piece with oneself [13,14,15,16,17,18]. Assessment of the spiritual needs of patients is complicated because of the ambiguity and complexity of the concept of spirituality and difficulties in differentiating between the concepts of religion and spirituality, especially when assessing spirituality in patients who are not religious.

In Lithuania, the present Law of the health system indicates spiritual wellbeing as a dimension of health, together with physical and social wellbeing of a person and society [19]. Nevertheless, in clinical practice, there is some confusion among healthcare providers who should be seen as responsible for providing spiritual care. The professional standards of practice for most doctors neglects the spiritual dimension in healthcare with a few exceptions only, i.e., oncologists, palliative care providers, and physician anesthesiologist-intensivist [20,21]. This is in accordance with the standards of supportive treatment methods and with the principals of palliative. The standard for a general practice nurse (2011) requires that they are able to assess patient’s needs by a comprehensive creating care plan. However, inclusion of spiritual aspects may be supposed as relying only on the content of nursing curriculum where holistic care and all possible dimensions of it are emphasized during training of nurses to assess, meet, and evaluate patient’s needs [22].

Like in other countries [23,24], in Lithuania, there are intentions to create a model of spiritual support provision in healthcare institutions, expanding pastoral care in hospitals, and actualizing the figure of the hospital chaplain by clarifying his role and duties in supporting patients and their families during moments of great emotional, spiritual, and psychosocial loss and grief [25]. On the other hand, professional spiritual care should not be limited to clergy staff visits under requests. Healthcare professionals are expected to work as multidisciplinary teams, and spiritual needs of patients have to be assessed and supported by the whole team relying on competences of each member.

To our knowledge, no significant research on the topic of spiritual needs has been, so far, developed in Lithuania (as a former state of the Soviet Union). There have been no validated measures of spiritual needs for use in clinical settings in Lithuania as well. The selection of culturally and structurally relevant tools was based on the analysis of the literature and review of similar tools: Spiritual interests related to illness tool (SpIRIT) by Taylor [13], spiritual needs scale (SNS) by Yong et al. [26], spiritual needs questionnaire (SpNQ) by Büssing et al. [27], spiritual needs assessment for patients questionnaire (SNAP) by Sharma et al. [28], functional assessment of chronic illness therapy—spiritual wellbeing scale (FACIT-Sp) by Canada et al. [29], spiritual needs questionnaire for palliative care by Vilalta et al. [30], spiritual care needs inventory (SCNI) by Wu et al. [31], and others.

The choice we made on spiritual needs questionnaire (SpNQ) is explained, firstly, by European nature of the instrument as it was developed in 2009 in Germany. Secondly, the tool was developed to measure the intensity of a person’s unmet psychosocial, existential, and spiritual needs in a standardized way [27,32]. Thirdly, the tool was designed for its use in adults with chronic diseases and suitable for interview administration (beside self or telephone administration). Fourthly, previous qualitative interviews with clinicians, social workers, psychologists, members of clergy, and patient relatives provided the information about the main features of cancer patients spiritual needs while in hospital. Fifth, the instrument is available in different language versions with quite stable factorial structure, and thus allows cultural comparisons, too (www.spiritualneeds.net). Further, the underlying theoretical basis for the SpNQ refers to four core dimensions of spiritual needs, i.e., connection, peace, meaning/purpose, and transcendence [14]. These are divided into categories of social, emotional, existential, and religious needs. Finally, SpNQ is intended to collect the information on patient’s spiritual concerns while also being simple in language and content, to start communication with patients about their spirituality and their unmet needs and thus enabling to develop spiritual care plan to address their unmet needs. The SpNQ was translated and validated in the different languages and used in the following countries: Germany [6,27,33,34], Portugal [35], Poland [36], China [37], Croatia [38], Pakistan [39], Brazil [35], Indonesia [40,41], Iran [42,43], etc. Such wide application of the tool, apart from in Anglo-Saxon countries, is usually promising for successful instrument validation in different culture and language.

In this study, we aimed to translate, adapt, psychometrically test, and validate the spiritual needs questionnaire in the Lithuanian language, culture, and healthcare practice.

## 2. Materials and Methods

### 2.1. Study Design and Methods

A descriptive, cross-sectional survey design was used for this study. Structural individual interview method (face-to-face) was employed to collect data on spiritual needs of hospitalized cancer patients. Before data collection, the principal researcher provided sufficient consultations with the interviewers regarding instructions for completing the instrument, the specific language, and the meaning of terms. Trained interviewers (two nurses and two final year undergraduate nursing students) visited the hospital and administered the questionnaire on a one-to-one basis, at the most convenient time for patients, in a calm and private place, for an average duration of 40 min (min 15, max 80), depending on the health status and the age of the respondent. The managers directed the researchers to patients that met the inclusion criteria.

### 2.2. Participants

In order to assess spiritual needs of cancer patients, responses were obtained from 247 patients hospitalized in nursing and supportive treatment units at public hospitals.

There were more female patients (60.7%) than male. The age of patients varied from 32 years to 96 years, and the mean age was 67.14 ± 11.62. Two-thirds (78.4%) identified themselves as religious persons, 10.2% as non-religious, and 11.4% were undecided.

### 2.3. Study Instrument

The underlying theoretical basis for the SpNQ refers to four core dimensions of spiritual needs, i.e., connection, peace, meaning/purpose, and transcendence [27]. The questionnaire uses 20 to 27 items (plus 3 free text fields) and differentiates four main factors: Religious needs, needs for inner peace, existential needs (including forgiveness needs), and giving/generativity needs. In the current version of the SpNQ, that is widely used internationally, 20 items compose the four need scales and seven items are informative only and are not used in the scales. All SpNQ items score with respect to the self-ascribed importance (‘intensity’) on a 4-point scale from disagreement to agreement (0—not at all; 1—somewhat; 2—very; 3—extremely). The higher the scores, the stronger the patient’s respective needs are.

For this paper we report on the translation and validation process of SpNQ Lithuanian version that contains 27 items (SpNQ-27). Additionally, with the perspective of international data comparison, we tested construct validity of SpNQ Lithuanian version that uses the same 20 items as the current version of the SpNQ-20 [44].

### 2.4. Translation Procedures

The SpNQ-27 was translated following methodological considerations [45]. A nurse educator and last-year bachelor nursing student, as two native, local culture and language translators, accomplished the initial translation of the instrument from English language to the Lithuanian language.

Later, both the translated Lithuanian versions were compared and discussed, and a consensus reached, taking into account the principles of instrument translation/adaptation. The choice of experts with relevant expertise, e.g., spiritual counseling, knowledge of theology, and proficiency in Lithuanian language, were considered to deal with linguistic and cultural differences in translation. In addition to that, for item wording and meaning clarification purposes, Polish and German versions were revised.

English language specialists conducted back translation of the agreed Lithuanian version of SpNQ-27 questionnaire. Achievement of equivalency and congruence was achieved between the original and translated versions of the instrument when comparing both English versions (original and back translated) and compared with the primary German language version. This task was accomplished by the primary author of the instrument (Büssing, A.). Any discrepancies were discussed among the researchers, the author, and the Lithuanian native English language philologist in seeking the greatest agreement. The Lithuanian language style, syntax, and grammar were corrected by a language specialist several times during the translation procedure.

### 2.5. Ethical Considerations

The Lithuanian Regional Committee on Bioethics issued permission to conduct the study (5 December 2017, No. BE-2-84). Cancer patients received written information about the aim of the study and signed informed consent. All questionnaires were with codes and participant (patient or student) identifying information was not available.

### 2.6. Statistical Analysis

Data were recorded and analyzed using the Statistical Package for Social Sciences (IBM SPSS Statistics, Armonk, NY, IBM Corp.) version 22.0. To assess the psychometric properties of the scale, Cronbach’s alpha, split half test, average inter-item, and item-total correlations were calculated for internal consistency. Items scoring below 0.15 have poor inter-item correlations, suggesting that they are not that well related to each other. Items that correlate above 0.50 tend to be very similar to each other. Cronbach’s alpha coefficient was calculated for individual factors and the whole scale; the internal consistency of α > 0.6 was considered to be acceptable [46]. The Spearman’s rank correlation coefficient (rho) (between the spiritual needs domain) scores produced at the first and the second testing calculated to assess the test-retest reliability.

Exploratory factor analysis was used to compare the extracted latent factors of the translated version of SpNQ with the construct of original version of SpNQ. Principal component analysis with varimax rotation was applied. The Kaiser-Meyer-Olkin and Bartlett’s test of sphericity were used to assess the appropriateness of the sample for the factor analysis. Eigen values > 1 and the scree plot were used to determine the number of factors.

To test the stability and reliability of an instrument over time, the test-retest reliability method was used. The Spearman’s rank correlation coefficient (rho) between four domains scores produced at the first and the second testing was calculated to assess the test-retest reliability (the Pearson’s correlation coefficient was calculated for the total score because of normality of scores in both testing’s). With respect to the correlation analysis, we regarded r > 0.5 as a strong correlation, 0.3 < r < 0.5 as a moderate correlation, 0.2 < r < 0.3 as a weak correlation, and r < 0.2 as no or a negligible correlation.

## 3. Results

### 3.1. Face and Content Validity of the SpNQ-27 Lithuanian Version

The advantage of SpNQ items is that the majority uses short statements in clear lexis starting with the verb form. This is much easier for ill patients to get the sense of what is being asked rather than listening to long sentences with complicated syntactic constructs. Instrument applicability and comprehensiveness was taken into consideration during the translation process. Expressions such as open aspects of your life, loving attitude, higher presence, and being complete and safe were difficult to find culturally and linguistically appropriate literal equivalents that would be familiar to the Lithuanian patients. 

Discussion between the Lithuanian researchers and the author of SpNQ (Büssing, A.) provided a wider exploration of meaning, which led to an accurate interpretation and avoidance of semantic errors. For example, the word *worries* has several meanings in Lithuanian language: Concerns, troubles, and anxiety; although according to the comments of the author, anxiety was avoided in translation as it was not appropriate. Another example of exact meaning clarification was with the item that ‘someone of your religious community (i.e., pastor) cares for you’. The word *care* was an issue as the meaning includes much more than a simple visit. Another manifestation of instrument translation challenge was the item ‘to dwell at a place of quietness and peace’. In Lithuanian, it was translated as ‘to get your mind into a quiet and peaceful place’, keeping in mind hospitalized patients or ill persons that have limited mobility. Only the equivalence checking revealed this incongruence in translation as it was originally meant to be personally in such inspiring places of quietness and peace, not mentally only. The item ‘to give away something from yourself’ requested additional clarification on what things, material or non-material, were in mind. In the item ‘for being complete and safe’, the word *complete* was confusing until explained as to be in complete health and wellbeing. The spiritual issues always relate to emotions and deep subjective meaning [1] and to reflect the intended emotion the strength of verb or adjective may be important in asking a particular question. For example, in our case, the item ‘to plunge into beauty of nature’ in the primary translation communicated ‘to admire the nature’; at the equivalence checking stage, the verb *admire* was noted as being too weak to express the original meaning, i.e., the feeling to be part of the nature, to ‘dissolve’ in nature.

Further, to ensure that item content had similar meaning for the intended population, the pilot testing was conducted with cancer patients during the first three weeks of the survey.

### 3.2. Test Re-Test Protocol

To test the reliability (stability in time) of the Lithuanian versions of the SpNQ-27, a test-retest study was arranged when third-year bachelor of nursing students (N = 40) completed the questionnaire twice, within one week of the initial test and subsequent re-test. The clarity of the instrument was discussed with students. The reliability of the questions based on the agreement of nursing students’ responses was investigated. For SpNQ-27 and, separately for SpNQ-20, the Pearson’s correlation coefficient calculated for the total score because of normality of scores in both tests. The total SpNQ-27 correlation coefficient was 0.88. The total SpNQ-20 correlation coefficient was 0.87.

In relation to SpNQ-20 domains, Spearman’s rank correlation of Religious needs was 0.84, for existential needs was 0.78, for giving/generativity and forgiveness needs was 0.73, and Pearson correlation for inner peace needs was 0.84.

#### Construct Validity of the SpNQ-27 Lithuanian Version

The goal of factor analyses was to determine the structure of the longer SpNQ-27 Lithuanian version. The Kaiser-Meyer-Olkin (KMO) values (0.927) indicated that data and sample size were adequate for factor analysis. Moreover, the approximate Chi-square values of Bartlett’s test of sphericity (χ^2^ (351) = 3305,092, *p* < 0.001) confirmed that the factor model is appropriate. These two tests showed the suitability of the respondent data for exploratory factor analysis, which was performed on the SpNQ-27 items using the principal component factor analysis with Varimax rotation. Items loaded significantly on four factors:

- Religious needs: This factor was composed of nine items and obtains loads between 0.471 and 0.824 factorials. Item N2 loaded weakly on the religious needs factor and the other two factors: Existential and family support needs factor and giving/generativity and forgiveness needs factor.
- Giving/generativity (and forgiveness): This factor was comprised of seven items with factorial loads ranging from 0.452 to 0.774. Item N17 loaded weakly on both the giving/generativity and forgiveness needs factor and the religious needs factor. Similarly, item N10 loaded weakly on both the giving/generativity and forgiveness needs factor and the inner piece factor.
- Inner peace needs: This factor was composed of five items with factorial loads ranging from 0.430 to 0.783. Item N5 loaded weakly on both the inner peace needs factor and the giving/generativity and forgiveness needs factor.
- Existential and connectedness with family needs: This factor contained six items with factorial loads between 0.379 and 0.713. The relatively new items about relationship with family and family support that were not primarily used in SpNQ-20 version now loaded in this category. Item N24 loaded weakly on both existential and family support needs factor and inner piece needs factor.

**Figure 1:**
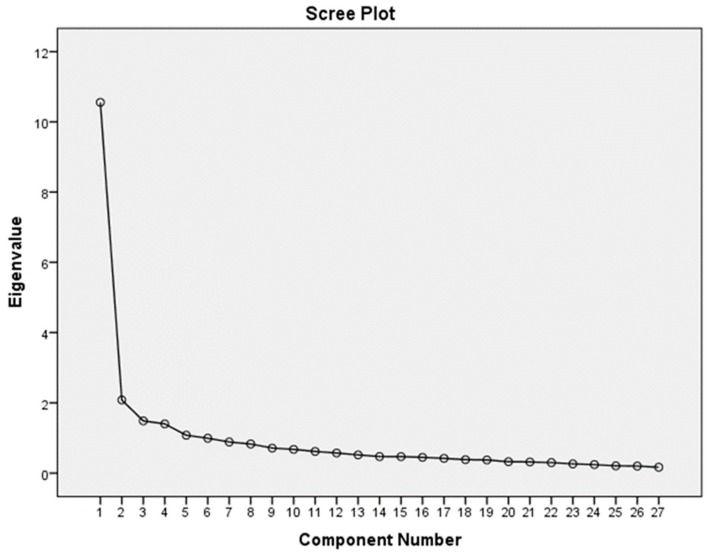
Scree plot of Lithuanian SpNQ-27 version.

These four factors had an eigenvalue greater than 1 with an explained variance of 57.5%. In total, 22 items out of 27 had a loading range higher than 0.5, above the minimum acceptable value of 0.4 (Table 1, Figure 1).

### 3.3. Reliability Analysis of SpNQ-27

#### 3.3.1. Internal Consistency

Lithuanian version of SpNQ-27 had a good internal consistency (Cronbach’s alpha = 0.94). The existential and connectedness with family needs factor had the lowest Cronbach’s alpha (0.71) in relation to other factors: Religious needs (0.93), giving/generativity and forgiveness needs (0.88) and inner peace needs (0.74).

Split-half test showed strong relationship between the both halves of the test (Spearman-Brown coefficient was 0.88).

The item difficulty (1.47 (mean value)/3) was 0.49; while all values were in acceptable range from 0.20 to 0.80.

Item-total correlations were inspected for the items in each of the four SpNQ-27 factors. For the religious needs factor, the corrected item-total correlation ranged from 0.56 to 0.80; the item-total correlation of this factor was the strongest in comparison with the other three factors. For the existential and connectedness with family needs factor, the corrected item-total correlation was the lowest and ranged from 0.23 to 0.59 (Table 1).

#### 3.3.2. Average Inter-Item Correlation

In the religious needs factor, the correlation coefficient ranged from 0.40 to 0.73; the giving/generativity and forgiveness needs factor correlation coefficients ranged from 0.41 to 0.69; in the inner peace needs factor, it ranged 0.20–0.56, and in the existential and connectedness with family needs factor correlation coefficients varied from 0.12 to 0.46.

Although the aim of this paper was limited to the reporting on instrument translation and validation, brief descriptive data on four spiritual needs domain of SpNQ-27 were calculated. Results revealed that the most important for non-terminally ill cancer patients were their unmet existential and connectedness with family needs (1.92 ± 0.72), giving/generativity and forgiveness needs (1.58 ± 0.65); inner peace needs (1.46 ± 0.72), while religious needs were of the least importance (1.23 ± 0.79), but nevertheless above 1.0, indicating relevance.

### 3.4. Construct Validity of the SpNQ-20 Lithuanian Version

For this analysis, we used only those 20 items that were confirmed in previous analyses of SpNQ as the final version [43]. This version does not use the needs to be connected/supported by the family. The Kaiser-Meyer-Olkin (KMO) values (0.92) indicated that data and sample size were adequate for factor analysis. Moreover, the approximate Chi-square values of Bartlett’s test of sphericity (χ^2^ (190) = 2509,151, *p* < 0.001) confirmed that the factor model is appropriate. These two tests showed the suitability of the respondent data for exploratory factor analysis, which was performed on the SpNQ-20 items using the principal component factor analysis with Varimax rotation. Items loaded significantly on four factors:

- Religious needs: This factor I was composed of eight items and obtained loads between 0.46 and 0.84 factorials. Item N2 loaded weakly on both the religious needs factor and factor II and factor III.
- Giving/generativity (and forgiveness) needs: This factor II was comprised of four items with factorial loads ranging from 0.61 to 0.78.
- Existential needs: This factor III contained five items with factorial loads between 0.42 and 0.71. Item N11 loaded weakly on both the existential needs factor and factors I and II.
- Inner peace needs: This factor IV was composed of three items with factorial loads ranging from 0.65 to 0.85.

**Figure 2:**
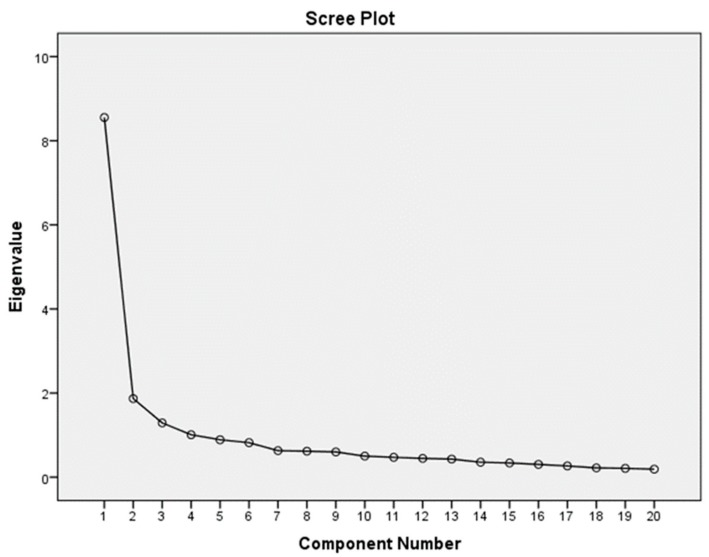
Scree plot of Lithuanian SpNQ-20 version.

These four factors had an eigenvalue greater than 1 with an explained variance of 63.6%. In total, 18 items out of 20 had a loading range higher than 0.5, above the minimum acceptable value of 0.4 (Table 2, Figure 2).

### 3.5. Reliability Analysis of SpNQ-20

#### 3.5.1. Internal Consistency

Lithuanian version of SpNQ-20 had a good internal consistency (Cronbach’s alpha = 0.93). The inner peace needs factor had the lowest Cronbach’s alpha (0.71) in relation to other factors: Religious needs (0.92), giving/generativity needs (0.84), and existential needs (0.77).

Split-half test showed strong relationship between the both halves of the test (Spearman-Brown coefficient was 0.85).

Item-total correlations were inspected for the items in each of the four SpNQ-20 factors. For the religious needs factor, the corrected item-total correlation ranged from 0.56 to 0.79 (Table 2).

#### 3.5.2. Average Inter-Item Correlation

In the religious needs factor, the correlation coefficient ranged from 0.40 to 0.73; the giving/generativity needs factor correlation coefficients ranged from 0.47 to 0.70; in the inner peace needs factor, the range was 0.38–0.56; and in the existential needs factor, correlation coefficients varied from 0.30 to 0.55.

## 4. Discussion

Research on the phenomenon of spirituality in healthcare is growing internationally. Each clinician is responsible for approaching the patient as a whole person and to provide relational, dignity-based, compassionate care [3]. Holistic care requires that spiritual needs of patients would be addressed and satisfied by healthcare professionals [17] in collaboration with social care and clergy staff and patient relatives.

Importantly, for rigorous spirituality assessment, well-prepared, valid, and reliable measurement tools are needed. The development of new research tool is always a time-consuming process requiring the involvement of competent investigators and experts of the field. For this reason, instead of developing their own study instruments, researchers more often search and select the most appropriate already existing tool to perform its linguistic, cultural, and practical adaptation. The aim of this study was to translate and validate the SpNQ for its use in the Lithuanian context.

The original version of SpNQ-20 [27,47] addresses a wide range of spiritual needs, both private and institutional religiosity (i.e., praying, congregational activities, reading spiritual/religious books, involvement of chaplains, etc.); forgiveness; existentialistic issues in terms of life reflection and meaning of life and suffering; social interactions, attention by others, and active compassionate turning to others; the need for inner peace and beauty of nature, etc. [27]. The topic of family support or family connection needs was part of the original item pool, but primarily not included in the factorial structure, because these needs might be considered as psychosocial. Nevertheless, in Brazil, these needs were considered as being very important [35] and thus, for the Lithuanian SpNQ-27 version, these items were included, too. There is evidence that good family support is linked to effective coping strategies and lower anxiety levels in cancer patients [48]. The orientation toward ‘cancer as communal’ is beneficial in a sense of relational and communication opportunities that family may provide [49]. Moreover, family support is perceived as a main spiritual need of cancer patients by clinical nurses as well [50].

The quality of research instrument adaptation heavily relies on its proper translation. At this stage, the main issues arise because of linguistic and semantic peculiarities of languages and differences in grammar, syntax, and lexis. Lithuanian language is related to Latvian and dead Prussian. It is important to note that as a separate branch of Eastern Baltic languages, the Lithuanian language started to be developed from the seventh century and it still retains ancient grammar forms and morphology such as in Latin or ancient Greek. By comparison, ‘English belongs to analytic language and Lithuanian is considered as a synthetic language’ [51]. Word order, cases, gender, and number in Lithuanian sentences differ from English sentence structure. Lithuanian grammar rules considering word order are less strict than English ones. The change of word order in the English sentence usually alters the meaning of the whole sentence, while word order in Lithuanian sentence is free. Since English and Lithuanian languages have their unique peculiarities in sayings and phrases, it causes quite a few problems to translate from one language into another without losing the exact meaning [52]. All these aspects were influential during the translation of SpNQ from English language to Lithuanian.

The strict methodological plan and right human resources during translation process assured good comprehensiveness of the instrument. A conversational approach with respondents being questioned via face-to-face interviews by two final year nursing students allowed us to determine if the respondent had any difficulty in understanding any items because of personal features or health condition (older age, poor health literacy, weak health status). Interviewers reflected that the application of tool was rather quick in time, without any repeatable obscurities in item wording.

Validation processes of Lithuanian version SpNQ-27 included the four-factor structure, similar to original, for spiritual needs: Religious needs, existential and connectedness with family needs, inner peace needs, and giving/generativity and forgiveness needs. However, the diversity of translated versions caused some doubt in choosing the model as validation of instrument in other countries used a five factor scale: Religious needs, existential needs, inner peace needs, giving/generativity and forgiveness needs, and adding an additional (independent) category called family support needs [35]. Also, the Croatian [38] version of SpNQ-23 used an additional “non-spiritual” category—social support needs.

Both Lithuanian versions of SpNQ demonstrated adequate psychometric properties of the instrument. Among the 27 tested SpNQ items, the construct had a good internal consistency (Cronbach’s alpha = 0.94), the same as among 20 tested SpNQ items (Cronbach’s alpha = 0.93). Similar results were demonstrated in the Chinese version [37] where among 20 tested items, internal consistency alpha was 0.82; in the Polish version, internal consistency alpha among 18 tested items was 0.89 [36].

Having the intention to compare the Lithuanian data on spiritual needs internationally, the psychometric properties of SpNQ-20 Lithuanian version were measured accordingly. Factor analysis on the Lithuanian version of SpNQ-20 revealed slight differences in relation to the original German version of measurement. Exploratory factor analysis of the original version of SpNQ [27,44] pointed out that the religious needs factor (eigenvalue 8.6; alpha = 0.90) consists of six items (N18, N19, N20, N21, N22, N23). Similar results with the same six items on the religious needs domain were found in the Polish version of SpNQ [36]. The religious need domain of the Lithuanian version of SpNQ-20 had the same six items as the German and Polish versions, but an additional two items (N2—talk with someone about fears and worries and N12—talk about the possibility of life after death) were bracketed together for this domain.

In the Lithuanian version of SpNQ-20, three items (N6, N7, N8) composed the inner peace needs factor and this structure corresponds with the original German version [44], although the original version of inner peace had four items, while in the Lithuanian SpNQ-20 version, one item (N2) about sharing fears and worries with someone was transferred to the religious needs domain. However, because of very low loading, it did not fit any of four factors and should be removed from the survey tool at the data analysis stage.

The major differences in the factor analysis of the Lithuanian version of SpNQ-20 were found in giving/generativity and forgiveness needs and the existential needs domains. Original SpNQ domains giving/generativity and forgiveness needs [44] consists of four items (N 14, N15, N26, N27). In the Lithuanian version, the items N14 and N15 coincident with original German version, although two other items were added (N16, N17). These two abovementioned items reflect forgiveness and in the original German version belong to existential needs factor.

In the German version of SpNQ [44], the existential needs domain had six items (N5, N10, N11, N12, N16, N17), while among the 20 tested SpNQ items in Lithuanian version three items were the same (N5, N10 and N11), but the other two items (N26 and N27) included here originally describe giving/generativity needs.

Validation of the SpNQ in the Lithuanian language and clinical practice revealed that the domain of religious needs was the most stable and complied with original German version the best. On the contrary, existential needs as well as giving/generativity and forgiveness needs domains in Lithuanian versions of SpNQ-27 and SpNQ-20 lost their original structure at the most extent. The existential items describing needs of forgiveness in both Lithuanian versions of the instrument were associated with the giving/generativity and forgiveness needs. The item switch often happens due to cultural differences and the way things are interpreted by populations in different contexts. For this reason, careful attention should be paid to contextual features more generally in the translation and validation process of the spiritual needs questionnaire.

The validation process of the Lithuanian version of SpNQ has some limitations. Firstly, the instrument requires evidence for its concurrent validity, by comparing the findings with data from other sources and applying congruent or divergent measurement tools. Secondly, the Lithuanian version of SpNQ was only used with one homogenous sample of cancer patients, while the general SpNQ was validated in larger samples of different populations [44]. More variety would be advisable. Thirdly, repeated testing of psychometric properties is recommended for data collected by other means, such as independent responding.

## 5. Conclusions

The Lithuanian version of the spiritual needs questionnaire was translated and validated on cancer hospitalized patients, demonstrating adequate psychometric properties of the instrument. The longer version of the spiritual needs questionnaire is useful to estimate the family dimension of patients’ unmet spiritual needs in the Lithuanian context where family continues to be a core value of individuals and society. Application of the Lithuanian version of SpNQ-20, which keeps items from the original structure, allows international comparison of the results and identification of cultural differences in patients’ perception of their spiritual needs.

The spiritual needs questionnaire, as a screening tool and conversational model, is recommended for clinicians in healthcare practice to identify patients with spiritual needs and plan their holistic care. 

## Figures and Tables

**Table 1:** Factorial structure of the Lithuanian version of spiritual needs questionnaire—SpNQ-27.

Item	Mean Value	Standard Deviation	Difficulty Index (0.49)	Corrected Item-Total Correlation	α If Item Deleted	Factors			
						I	II	III	IV
**Religious Needs (Eigenvalue 10.56; α = 0.927)**									
N18. Pray with someone	1.12	1.04	0.37	0.777	0.916	**0.824**			
N21. Participate at a religious ceremony (i.e., service)	1.34	1.04	0.45	0.803	0.914	**0.806**			
N19. Someone prays for you	1.31	1.01	0.44	0.781	0.915	**0.796**			
N3. That someone of your religious community (i.e., pastor) cares for you?	0.98	1.03	0.33	0.746	0.918	**0.785**			
N22. Read religious/spiritual books	1.00	1.03	0.33	0.764	0.916	**0.767**			
N20. Pray for yourself	1.53	1.00	0.51	0.798	0.914	**0.747**			
N23. Turn to a higher presence (i.e., God, Angels, etc.)	1.43	1.04	0.48	0.769	0.916	**0.722**			
N12. Talk about the possibility of life after death	0.82	0.86	0.27	0.596	0.926	**0.565**	0.345		
N2. Talk with someone about fears and worries	1.28	0.96	0.43	0.557	0.929	0.471	0.376	0.372	
**Giving/Generativity and Forgiveness Needs (Eigenvalue 2.06; α = 0.879)**									
N15. Give solace to someone	1.57	0.92	0.52	0.718	0.855		**0.774**		
N14. Give away something from yourself	1.51	0.97	0.50	0.690	0.858		**0.692**		
N16. Forgive someone from a distinct period of your life	1.62	0.89	0.54	0.669	0.861		**0.662**		
N13. To turn to someone in a loving attitude	1.60	0.83	0.53	0.684	0.860	0.311	**0.657**		
N11. Talk about the question of meaning in life	1.29	0.90	0.43	0.660	0.862	0.355	**0.562**	0.364	
N17. Be forgiven	1.58	0.97	0.53	0.657	0.863	0.396	0.485		
N10. Find meaning in illness and/or suffering	1.42	0.97	0.47	0.577	0.873		0.452	0.368	
**Inner Peace Needs (Eigenvalue 1.49; α = 0.739)**									
N7. Dwell at a place of quietness and peace	2.11	0.91	0.70	0.489	0.699			**0.783**	
N8. Find inner peace	1.96	0.88	0.65	0.589	0.661			**0.678**	
N6. Plunge into beauty of nature	1.69	0.93	0.56	0.487	0.700			**0.655**	
N4. Reflect on previous events in life	1.48	0.80	0.49	0.500	0.696	0.319		**0.542**	
N5. Dissolve open aspects of your life	1.48	0.90	0.49	0.451	0.713		0.326	0.430	
**Existential and Connectedness with Family Needs (Eigenvalue 1.40; α = 0.710)**									
N28.To be re-involved by your family in their life concerns	2.12	0.80	0.71	0.589	0.625				**0.713**
N25.To feel connected with family	2.35	0.67	0.78	0.507	0.658				**0.651**
N26. Pass own life experiences to others	1.75	0.90	0.58	0.511	0.648		0.387		**0.633**
N29. To receive more support from your family	1.89	1.03	0.63	0.410	0.691				**0.569**
N27. Assured that your life was meaningful and of value	1.75	0.78	0.58	0.446	0.670		0.383		**0.543**
N24. For being complete and safe	2.55	0.66	0.85	0.232	0.724			0.304	0.379

Principal component analysis (Varimax rotation with Kaiser normalization); the respective four factors would explain 57.5% of variance; factor loadings < 0.3 are not depicted; factor loadings greater than 0.5 are in bold type.

**Table 2:** Factorial structure of the Lithuanian version of spiritual needs questionnaire—SpNQ-20.

Item	Corrected Item-Total Correlation	α If Item Deleted	Factors			
			I	II	III	IV
**Religious Needs (Eigenvalue 8.55; α = 0.917)**						
N21. Participate at a religious ceremony (i.e., service)	0.793	0.900	**0.835**			
N19. Someone prays for you	0.785	0.901	**0.822**			
N18. Pray with someone	0.754	0.903	**0.816**			
N22. Read religious/spiritual books	0.755	0.903	**0.789**			
N20. Pray for yourself	0.789	0.900	**0.776**			
N23. Turn to a higher presence (i.e., God, Angels, etc.)	0.765	0.902	**0.744**	0.356		
N12. Talk about the possibility of life after death	0.590	0.916	**0.578**		0.362	
N2. Talk with someone about fears and worries	0.561	0.919	0.457	0.345	0.320	
**Giving/Generativity and Forgiveness Needs (Eigenvalue 1.87; α = 0.836)**						
N15. Give solace to someone	0.705	0.776		**0.778**		
N16. Forgive someone from a distinct period of your life	0.671	0.792		**0.752**		
N14. Give away something from yourself and of value	0.687	0.784		**0.706**		
N17. Be forgiven	0.609	0.819	0.398	**0.611**		
**Existential Needs (Eigenvalue 1.01; α = 0.769)**						
N5. Dissolve open aspects of your life	0.573	0.714			**0.708**	
N26. Pass own life experiences to others	0.505	0.738			**0.685**	
N27. Assured that your life was meaningful	0.446	0.756			**0.642**	
N10. Find meaning in illness and/or suffering	0.592	0.707	0.312		**0.548**	0.301
N11. Talk about the question of meaning in life	0.581	0.711	0.364	0.418	0.423	
**Inner Peace Needs (Eigenvalue 1.29; α = 0.708)**						
N7. Dwell at a place of quietness and peace	0.554	0.581				**0.852**
N8. Find inner peace	0.580	0.550				**0.721**
N6. Plunge into beauty of nature	0.447	0.715				**0.652**

Principal component analysis (Varimax rotation with Kaiser normalization); the respective four factors would explain 63.6% of variance; factor loadings < 0.3 are not depicted; factor loadings greater than 0.5 are in bold type.

## References

[1] Caldeira S., Romeiro J., Martins H., Casaleiro T. (2019). The therapeutic dimension of research about spirituality: Particularities of cancer, mental health and infertility. Nurs. Forum.

[2] Hellman A., Williams W., Hurley S. (2015). Meeting Spiritual Needs: A Study Using the Spiritual Care Competence Scale. J. Christ. Nurs..

[3] Balboni M.J., Puchalski C.M., Peteet J.R. (2014). The relationship between medicine, spirituality and religion: Three models for integration. J. Relig. Health.

[4] Sulmasy D.P. (2009). Spirituality, Religion, and Clinical Care. Chest.

[5] Büssing A., Janko A., Baumann K., Hvidt N.C., Kopf A. (2013). Spiritual Needs among Patients with Chronic Pain Diseases and Cancer Living in a Secular Society. Pain Med..

[6] Offenbaecher M., Kohls N., Toussaint L.L., Sigl C., Winkelmann A., Hieblinger R., Walther A., Büssing A. (2013). Spiritual needs in patients suffering from fibromyalgia. Evid.-Based Complement. Altern. Med..

[7] Balboni T., Balboni M., Paulk M.E., Phelps A., Wright A., Peteet J., Block S., Lathan C., VanderWeele T., Prigerson H. (2011). Support of cancer patients’ spiritual needs and associations with medical care costs at the end of life. Cancer.

[8] Sprung C.L., Cohen S.L., Sjokvist P., Baras M., Bulow H.H., Hovilehto S., Ledoux D., Lippert A., Maia P., Phelan D. (2003). End-of-life practices in European intensive care units: The Ethicus Study. JAMA.

[9] Edwards A., Pang N., Shiu V., Chan C. (2010). The understanding of spirituality and the potential role of spiritual care in end-of-life and palliative care: A meta-study of qualitative research. Palliat. Med..

[10] Gergianaki I., Kampouraki M., Williams S., Tsiligianni I. (2019). Assessing spirituality: Is there a beneficial role in the management of COPD?. NPJ Prim. Care Respir. Med..

[11] Büssing A., Ostermann T., Koenig H.G. (2007). Relevance of spirituality and religion in German patients with chronic diseases. Int. J. Psychiatry Med..

[12] Selman L.E., Brighton L.J., Sinclair S., Karvinen I., Egan R., Speck P., Powell R.A., Deskur-Smielecka E., Glajchen M., Adler S. (2018). Patients’ and caregivers’ needs, experiences, preferences and research priorities in spiritual care: A focus group study across nine countries. Palliat. Med..

[13] Taylor E.J. (2006). Prevalence and associated factors of spiritual needs among patients with cancer and family caregivers. Oncol. Nurs. Forum.

[14] Büssing A., Koenig H.G. (2010). Spiritual Needs of Patients with Chronic Diseases. Religions.

[15] Büssing A., Michalos A.C. (2014). Spiritual Needs of Those with Chronic Diseases. Encyclopedia of Quality of Life and Well-Being Research.

[16] Hatamipour K., Rassouli M., Yaghmaie F., Zendedel K., Majd H.A. (2015). Spiritual needs of cancer patients: A qualitative study. Indian J. Palliat. Care.

[17] Forouzi M.A., Tirgari B., Safarizadeh M.H., Jahani Y. (2017). Spiritual Needs and Quality of Life of Patients with Cancer. Indian J. Palliat. Care.

[18] Phenwan T., Peerawong T., Tulathamkij K. (2019). The Meaning of Spirituality and Spiritual Well-Being among Thai Breast Cancer Patients: A Qualitative Study. Indian J. Palliat. Care.

[19] (1994). Law of the Health System of the Republic of Lithuania (in Lithuanian). http://www3.lrs.lt/pls/inter3/dokpaieska.showdoc_l?p_id=259520&p_query=&p_tr2=.

[20] The Standard of Doctor Oncologist Chemotherapy (in Lithuanian). The Order of Health Care Minister of the Republic of Lithuania on the Standard of Medicine of Lithuania MN 121:2018 Doctor Chemotherapy Oncologist Official Gazette, 2018-07-19, No. 821. https://e-seimas.lrs.lt/portal/legalAct/lt/TAD/TAIS.303922/asr.

[21] The Standard of Physician Anesthesiologist-Reanimathologist (in Lithuanian). The Order of Health Care Minister of the Republic of Lithuania on the Standard of Medicine of Lithuania MN 1054:2018 Physician Anesthesiologist-Reanimathologist. Rights, Functions, Competence and Responsibility, (Official Gazette, 2019-04-24, No. V-493). https://www.e-tar.lt/portal/lt/legalAct/1e09f9e0674711e9917e8e4938a80ccb.

[22] The Standard of Practice for a Nurse of General Practice (in Lithuanian). The Order of Health Care Minister of the Republic of Lithuania on the Standard of Medicine of Lithuania MN 28:2004 Nurse of General Practice: Rights, Functions, Competence and Responsibility, the order No. V-591, 08 June 2011. Official Gazette, 2011-06-14, No. 72-3490. https://e-seimas.lrs.lt/portal/legalAct/lt/TAD/TAIS.401304.

[23] Meel L. (2018). Defining the Context for Best Practices: Institutional Setting for Clinical Pastoral Care in Estonia. J. Relig. Health.

[24] Proserpio T., Piccinelli C., Clerici C.A. (2011). Pastoral care in hospitals: A literature review. Tumori.

[25] Luft J.P. (2016). Spiritual Care and CPE: 2nd Year Experience. J. Pastor. Care Couns..

[26] Yong J., Kim J., Han S.S., Puchalski C.M. (2008). Development and validation of a scale assessing spiritual needs for Korean patients with cancer. J. Palliat. Care.

[27] Büssing A., Balzat H.J., Heusser P. (2010). Spiritual needs of patients with chronic pain diseases and cancer-validation of the spiritual needs questionnaire. Eur. J. Med. Res..

[28] Sharma R.K., Astrow A.B., Texeira K., Sulmasy D.P. (2012). The Spiritual Needs Assessment for Patients (SNAP): Development and validation of a comprehensive instrument to assess unmet spiritual needs. J. Pain Symptom Manag..

[29] Canada A.L., Murphy P.E., Fitchett G., Peterman A.H., Schover L.R. (2008). A 3-factor model for the FACIT-Sp. Psycho-oncology.

[30] Vilalta A., Valls J., Porta J., Viñas J. (2014). Evaluation of spiritual needs of patients with advanced cancer in a palliative care unit. J. Palliat. Med..

[31] Wu L., Koo M., Liao Y., Chen Y., Yeh D. (2015). Development and Validation of the Spiritual Care Needs inventory for Acute Care Hospital Patients in Taiwan. Clin. Nurs. Res..

[32] Büssing A., Balzat H.J., Heusser P. (2009). Spirituelle Bedürfnisse von Patienten mit chronischen Schmerz-und Tumorerkrankungen. (In Germany). Perioper. Med..

[33] Höcker A., Krüll A., Koch U., Mehnert A. (2014). Exploring spiritual needs and their associated factors in an urban sample of early and advanced cancer patients. Eur. J. Cancer Care.

[34] Haußmann A., Schäffeler N., Hautzinger M., Weyel B., Eigentler T., Zipfel S., Teufel M. (2017). Religiöse/spirituelle Bedürfnisse und psychosoziale Belastung von Patienten mit malignem Melanom [Religious/Spiritual Needs and Psychosocial Burden of Melanoma Patients]. Psychother. Psychosom. Med. Psychol..

[35] Valente T.C.O., Cavalcanti A.P.R., Büssing A., Costa Junior C.P.C., Motta R.N. (2018). Transcultural Adaptation and Psychometric Properties of Portuguese Version of the Spiritual Needs Questionnaire (SpNQ) Among HIV Positive Patients in Brazil. Religions.

[36] Büssing A., Pilchowska I., Surzykiewicz J. (2015). Spiritual Needs of Polish patients with chronic diseases. J. Relig. Health.

[37] Büssing A., Zhai X.F., Peng W.B., Ling C.Q. (2013). Psychosocial and spiritual needs of patients with chronic diseases: Validation of the Chinese version of the Spiritual Needs Questionnaire. J. Integr. Med..

[38] Glavas A., Jors K., Büssing A., Baumann K. (2017). Spiritual needs of PTSD patients in Croatia and Bosnia-Herzegovina: A quantitative pilot study. Psychiatr. Danub..

[39] Kashif A., Kanwal Z. (2018). Translation, Cultural Adaptation of Spiritual Needs Questionnaire in Pakistan. Religions.

[40] Nuraeni A., Nurhidayah I., Hidayati N., Windani C., Sari M., Mirwanti R. (2015). Kebutuhan Spiritual pada Pasien Kanker [Spiritual Needs of Patients with Cancer]. J. Keperawatan Padjadjaran.

[41] Munirruzzaman M., Yuni Sapto E.R., Prasetyo A., Kebutuhan G.T. Spiritual Pada Pasien Gagal Ginjal Kronik Yang Menjalani Hemodialisis Di Ruang Hemodialisa Rsud Cilacap [Description of Spiritual Needs Level in Chronic Kidney Failure Patients Who Underwent Hemodialysis in the Hemodialisa Room of RSUD Cilacap]. Proceedings of the Management Communication in Health Team Collaboration of Giving High Alert for Patient Safety, STIKES Al-Irsyad Al-Islamiyyah Cilacap.

[42] Moeini B., Zamanian H., Taheri-Kharameh Z., Ramezani T., Saati-Asr M., Hajrahimian M., Amini-Tehrani M. (2018). Translation and Psychometric Testing of the Persian Version of the Spiritual Needs Questionnaire Among Elders with Chronic Diseases. J. Pain Symptom Manag..

[43] Hatamipour K., Rassouli M., Yaghmaie F., Zendedel K. (2018). Psychometric Properties of the Farsi Version of “Spiritual Needs Questionnaire” for Cancer Patients in Iran: A Methodological Study. Asian Pac. J. Cancer Prev..

[44] Büssing A., Recchia D.R., Koenig H.G., Baumann K., Frick E. (2018). Factor Structure of the Spiritual Needs Questionnaire (SpNQ) in Persons with Chronic Diseases, Elderly and Healthy Individuals. Religions.

[45] Maneesriwongul W., Dixon J.K. (2004). Instrument translation process: A methods review. J. Adv. Nurs..

[46] Bland J.M., Altman D.G. (1997). Statistics notes: Cronbach’s alpha. BMJ.

[47] Büssing A., Janko A., Kopf A., Lux E.A., Frick E. (2012). Zusammenhänge zwischen psychosozialen und spirituellen Bedürfnissen und Bewertung von Krankheit bei Patienten mit chronischen Erkrankungen. (In Germany). Spirit. Care.

[48] Sari D.K., Dewi R., Daulay W. (2019). Association Between Family Support, Coping Strategies and Anxiety in Cancer Patients Undergoing Chemotherapy at General Hospital in Medan, North Sumatera, Indonesia. Asian Pac. J. Cancer Prev..

[49] Koenig K.J., Castle K.M., Johnson A.Z., Cohen M.Z. (2019). Cancer as Communal: Understanding Communication and Relationships from the Perspectives of Survivors, Family Caregivers, and Health Care Providers. Health Commun..

[50] Evangelista C.B., Lopes M.E.L., Costa S.F.G., Abrão F.M.S., Batista P.S.S., Oliveira R.C. (2016). Spirituality in patient care under palliative care: A study with nurses. Esc. Anna Nery.

[51] Pažūsis L., Rosinienė G., Žemaitienė U. (2005). English Language Grammar (in Lithuanian).

[52] Naudziunaite A. Comparative Structures in English and Their Counterparts in Lithuanian. Bachelor Thesis (in English), Siauliai University, Siauliai 2013. https://pdfs.semanticscholar.org/d7c1/24f0606edd1b2248041125513c3b5dec4914.pdf.

